# High Contrast PET Imaging of GRPR Expression in Prostate Cancer Using Cobalt-Labeled Bombesin Antagonist RM26

**DOI:** 10.1155/2017/6873684

**Published:** 2017-08-10

**Authors:** Bogdan Mitran, Helge Thisgaard, Ulrika Rosenström, Johan Hygum Dam, Mats Larhed, Vladimir Tolmachev, Anna Orlova

**Affiliations:** ^1^Division of Molecular Imaging, Department of Medicinal Chemistry, Uppsala University, Uppsala, Sweden; ^2^PET & Cyclotron Unit, Department of Nuclear Medicine, Odense University Hospital, Odense, Denmark; ^3^Department of Clinical Research, University of Southern Denmark, Odense, Denmark; ^4^Division of Organic Pharmaceutical Chemistry, Department of Medicinal Chemistry, Uppsala University, Uppsala, Sweden; ^5^Science for Life Laboratory, Department of Medicinal Chemistry, Uppsala University, Uppsala, Sweden; ^6^Department of Immunology, Genetics and Pathology, Uppsala University, Uppsala, Sweden

## Abstract

High gastrin releasing peptide receptor (GRPR) expression is associated with numerous cancers including prostate and breast cancer. The aim of the current study was to develop a ^55^Co-labeled PET agent based on GRPR antagonist RM26 for visualization of GRPR-expressing tumors. Labeling with ^57^Co and ^55^Co, stability, binding specificity, and in vitro and in vivo characteristics of ^57^Co-NOTA-PEG_2_-RM26 were studied. NOTA-PEG_2_-RM26 was successfully radiolabeled with ^57^Co and ^55^Co with high yields and demonstrated high stability. The radiopeptide showed retained binding specificity to GRPR in vitro and in vivo. ^57^Co-NOTA-PEG_2_-RM26 biodistribution in mice was characterized by rapid clearance of radioactivity from blood and normal non-GRPR-expressing organs and low hepatic uptake. The clearance was predominantly renal with a low degree of radioactivity reabsorption. Tumor-to-blood ratios were approximately 200 (3 h pi) and 1000 (24 h pi). The favorable biodistribution of cobalt-labeled NOTA-PEG_2_-RM26 translated into high contrast preclinical PET/CT (using ^55^Co) and SPECT/CT (using ^57^Co) images of PC-3 xenografts. The initial biological results suggest that ^55^Co-NOTA-PEG_2_-RM26 is a promising tracer for PET visualization of GRPR-expressing tumors.

## 1. Introduction

Receptors for small regulatory peptides are often overexpressed in human malignant tumors. This observation has led to a number of studies exploring the opportunity for clinical application of peptide analogs in tumor diagnosis and therapy [1]. One promising approach is to target the gastrin releasing peptide receptor (GRPR) using bombesin analogs. High GRPR expression was documented in the early, androgen-dependent stages of prostate cancer (PCa), but not in the hyperplastic prostate. Therefore GRPR has emerged as an attractive target in PCa, a notoriously difficult cancer to diagnose and stage using conventional imaging techniques.

Bombesin (BN) is a 14-amino acid peptide that binds to GRPR with high affinity. Over the past decade, several derivatives of bombesin have been investigated in preclinical and clinical studies. Initially, the consensus was that agonists were superior to antagonists due to the rapid radioligand internalization. The high internalization was considered to be essential for optimal imaging because it provides efficient intracellular trapping of radiometal labels [2]. However, the strong physiological action and mitogenicity associated with BN-based GRPR-agonists [3] and the superior tumor uptake seen for somatostatin antagonists [4] led to a paradigm shift towards the use of potent GRPR antagonists in the development of BN analogs during recent years [5].

Previously, our group reported a high affinity GRPR antagonist based on RM26 (DPhe-Gln-Trp-Ala-Val-Gly-His-Sta-Leu-NH_2_) [6] conjugated to a 1,4,7-triazacyclononane-N,N′,N′′-triacetic acid (NOTA) chelator via a diethylene glycol (PEG_2_) spacer (NOTA-PEG_2_-RM26). This construct was initially radiolabeled with ^68^Ga and ^18^F [7, 8] for positron emission tomography (PET) imaging and ^111^In [7] for single photon emission computer tomography (SPECT) imaging. The ^68^Ga and ^111^In-labeled constructs showed favorable pharmacokinetic properties and high affinity to GRPR. In an attempt to further improve the biodistribution profile for potential clinical use, we have also explored the use of different radionuclide-chelator complexes. It is known that the combination of radionuclide and chelator could have a strong influence on the targeting properties of radiopeptides [9, 10]. For example, the somatostatin analog [^57^Co-(dotatoc)] had the highest reported affinity for somatostatin receptor subtype 2 (SSTR2) [11]. Our studies on PEG_2_-RM26 labeled with ^68^Ga and ^111^In using different macrocyclic chelators demonstrated that charge and structure of the radionuclide-chelator complex have a strong influence on binding affinity and liver uptake of the radioconjugates [12, 13].

One interesting finding from our studies was that the tumor-to-background ratios increased significantly over time up to 24 h after injection (pi) [7, 13] due to the high uptake and long retention in tumors in combination with the fast clearance from blood and GRPR-expressing organs, underscoring the importance of radionuclide half-life in high sensitivity molecular imaging. These data are in agreement with findings from the head-to-head evaluation of ^55^Co- and ^68^Ga-labeled GRPR agonist NOTA-AMBA. In this study, ^55^Co-NOTA-AMBA provided superior imaging contrast compared to the ^68^Ga-labeled counterpart because of the possibility of performing imaging at 24 h pi. However, both ^68^Ga- and ^55/57^Co-labeled NOTA-AMBA had a prolonged retention in GRPR-expressing tissues, typical for agonists [14]. This issue might be solved by the use of a high affinity GRPR antagonist.

Thus, the use of a long lived radionuclide for next day imaging may enable the detection of low abdominal lymph node involvement, which requires the highest possible sensitivity and is an ultimate goal in prostate cancer imaging [15, 16]. Labeling of RM26 with a long lived positron-emitting radionuclide could further increase diagnostic accuracy due to the inherent high sensitivity and imaging quantitation capability of PET compared to SPECT. For next day imaging, a positron-emitting radiometal with a half-life of 10–20 h would be optimal. Possible PET radionuclides for such purposes include ^64^Cu (*T*1/2 = 12.7 h), ^86^Y (*T*1/2 = 14.7 h), and ^55^Co (*T*1/2 = 17.5 h). Among them, ^55^Co (76%  *β*^+^) has a higher positron abundance compared to ^64^Cu and a better ratio between annihilation photons and coemitted gammas compared to ^86^Y, providing better image quality [17]. The 17.5 h half-life of ^55^Co not only allows for delayed imaging investigations, but also enables shipment to stand-alone PET scanners.

The goal of this study was to evaluate the tumor targeting properties of GRPR antagonist NOTA-PEG_2_-RM26 labeled with ^55^Co.

## 2. Materials and Methods

### 2.1. Radiochemistry

NOTA-PEG_2_-RM26 (RM26 = [D-Phe6, Sta13, Leu14] bombesin [6-14]) was synthesized by standard manual solid-phase peptide synthesis based on previously published procedures [7].

^57^Co cobalt chloride was purchased from PerkinElmer Sweden (Upplands Väsby, Sweden). ^55^Co was produced in-house as previously described [18] with an extra purification step added [19]. Buffers, including 0.2 M ammonium acetate, pH 5.5, and 0.2 M citric acid, were produced in-house from chemicals supplied by Merck (Darmstadt, Germany). Phosphate buffered saline (PBS), pH 7.5, 0.1 M was purchased from the National Veterinary Institute, SVA, Sweden. Buffers for labeling were purified from free metals contamination by Chelex 100 resin (Bio-Rad Laboratories, Hercules, CA).

Radiolabeling with ^57^Co was performed using 3.8–10 nmol (aq. 1 nmol/*μ*L) of NOTA-PEG_2_-RM26 buffered with 80 *μ*L ammonium acetate (0.2 M, pH 5.5). The mixture was incubated with ^57^Co cobalt chloride (10–50 MBq) for 30 min at 60°C. For ^55^Co labeling, 1.3 nmol of NOTA-PEG_2_-RM26 buffered with 80 *μ*L ammonium acetate (0.2 M, pH 5.5) was heated by microwave irradiation for 1 min at 850 W in a sealed vial using a conventional microwave oven (Samsung MW82N-B). To evaluate the labeling stability, ^57^Co-NOTA-PEG_2_-RM26 was incubated in the presence of 1000-fold molar excess of EDTA disodium salt (Sigma) for 1 h at RT. Labeling yield, radiochemical purity, and in vitro stability were analyzed using instant thin-layer chromatography (ITLC) strips (Agilent Technologies) eluted with citric acid (0.2 M, pH 2.0). In this system, free ^57/55^Co migrates with the eluent front (*R*_*f*_ = 1.0) and the radiolabeled construct stays on the application point (*R*_*f*_ = 0.0). The system was previously validated by sodium dodecyl sulfate polyacrylamide gel electrophoresis (SDS-PAGE) and radio-HPLC [7, 8].

### 2.2. In Vitro Studies

PC-3 human prostate cancer cells expressing GRPR were purchased form ATCC via LGC Promochem. The cells were cultured in RPMI-1640 media supplemented with 10% fetal calf serum, PEST (penicillin 100 IU/mL, streptomycin 100 *μ*g/mL), and 2 mM L-glutamine (all from Biochrom AG, Berlin, Germany). This medium is referred to as complete medium in the text. Trypsin-EDTA (0.05% trypsin, 0.02% EDTA in buffer; Biochrom AG) was used for cell detachment. All in vitro experiments were performed in triplicate.

The binding specificity of ^57^Co-NOTA-PEG_2_-RM26 was evaluated using PC-3 cells incubated with 1 nM ^57^Co-NOTA-PEG_2_-RM26 solution for 1 h at 37°C. In this experiment one set of dishes was preincubated for 10 min at RT with an excess (100-fold) of nonlabeled peptide before applying the radioactivity. After incubation, the cells were washed with serum-free media and detached with 0.5 mL trypsin-EDTA solution. Radioactivity of cells was measured in an automated gamma-counter (3-inch NaI(Tl) detector, 2480 WIZARD^2^, PerkinElmer) and presented as cell-associated percentage from added radioactivity.

Cellular processing of the conjugate was evaluated using PC-3 cells incubated with 1 nM of ^57^Co-NOTA-PEG_2_-RM26 at 37°C, 5% CO_2_. At predetermined time points (1, 2, 4, 8, and 24 h after the start of incubation) the medium from 3 dishes was discarded and the cells were washed with ice-cold serum-free medium. The membrane-bound and internalized radioactivity were collected using the acid wash method described earlier [7].

The half maximal inhibitory concentration (IC_50_) was determined for ^nat^Co-NOTA-PEG_2_-RM26 using the universal BN radioligand ^125^I-Tyr^4^-BBN (Perkin Elmer). For comparison, an additional experiment was performed in parallel for the previously evaluated ^nat^In⁡-NOTA- PEG_2_-RM26, under the same experimental conditions [7]. Briefly, cell monolayers were incubated with ^125^I-Tyr^4^-BBN (0.1 pmol) for 5 h at 4°C in the presence of increasing concentrations of ^nat^Co-loaded and ^nat^In⁡-loaded conjugates (0–1000 nM). After incubation, the cells were collected and the cell-associated radioactivity was measured in an automated gamma-counter. The IC_50_ values were calculated by nonlinear regression using GraphPad Prism software (GraphPad Software Inc.).

The binding kinetics were measured in real-time using LigandTracer Yellow Instruments (Ridgeview Instruments AB) at room temperature, as described earlier [20]. The uptake curves were recorded at 0.33 and 6 nM. After measuring the uptake curves, the ^57^Co-NOTA-PEG_2_-RM26-containing medium was replaced by fresh medium and the dissociation curve was monitored overnight. Data were collected and evaluated using TracerDrawer software (Ridgeview Instruments AB).

### 2.3. In Vivo Studies

The animal experiments were planned and performed in accordance with the national legislation on the protection of laboratory animals, and the study plans were approved by the local committee for animal research ethics. Groups of 3-4 mice were used per data point. Biodistribution studies were performed on female immunocompetent NMRI mice (weight: 30 ± 2 g), female Balb/c nu/nu (inoculated 10 million PC-3 cells/mouse 21 days before the experiment, weight 17 ± 2 g), and male inbred immunodeficient NOD-SCID mice (5 million PC-3 cells/mouse, 35 days before the experiment, 31 ± 2 g).

### 2.4. Biodistribution and In Vivo Binding Specificity in NMRI Mice

Female NMRI mice were injected with 45 pmol (100 *μ*L in PBS) ^57^Co-NOTA-PEG_2_-RM26 into the tail vein. The injected radioactivity was adjusted to 30 kBq per animal. To test the in vivo binding specificity of ^57^Co-NOTA-PEG_2_-RM26 to murine GRPRs, one group of animals was intravenously coinjected with 20 nmol of unlabeled peptide. The mice were euthanized at 30 min pi by intraperitoneal injection of Ketalar-Rompun solution (10 mg/mL Ketalar and 1 mg/mL Rompun; 20 *μ*L/g body weight). Blood samples were collected by cardiac puncture. Lung, liver, spleen, pancreas, stomach, small intestines, kidneys, muscle, bone, and the rest of intestines with their content were collected. The organs were weighed and their radioactivity content was measured in a gamma-counter. The data were corrected for background radiation. The organ uptake values were expressed as a percentage of injected dose per gram of tissue (% ID/g), except for the gastrointestinal tract and the remaining carcass, which were calculated as % ID per whole sample.

### 2.5. Biodistribution and In Vivo Binding Specificity in PC-3 Xenografted Mice

Tumor targeting specificity and biodistribution over time were studied on PC-3 xenografted female Balb/c nu/nu and male NOD-SCID mice, respectively. For the in vivo binding specificity experiment, the animals were injected with 45 pmol (20 kBq, 100 *μ*L in 0.1% BSA in PBS) of ^57^Co-NOTA-PEG_2_-RM26. An additional group of animals was coinjected with an excess of nonlabeled peptide (20 nmol). The mice were sacrificed at 3 h pi by intraperitoneal injection of Ketalar-Rompun solution (10 mg/mL Ketalar and 1 mg/mL Rompun; 20 *μ*L/g body weight). Other groups of mice were intravenously injected into the tail vein with 16.8 ± 3.6 pmol of ^57^Co-NOTA-PEG_2_-RM26 (55 ± 12 kBq, 100 *μ*L in 0.1% BSA in PBS). The mice were euthanized at 3 and 24 h pi by cervical dislocation. Tumors, blood, and other organs of interest were collected, weighed, and measured on radioactivity content in a gamma-counter. The data were corrected for background radiation. The organ uptake values were expressed as a percentage of injected dose per gram of tissue (% ID/g), except for the gastrointestinal tract and the remaining carcass, which were calculated as % ID per whole sample.

### 2.6. SPECT/CT Imaging Using ^57^Co-NOTA-PEG_2_-RM26

Mice bearing PC-3 xenografts were intravenously injected with 100 pmol of ^57^Co-NOTA-PEG_2_-RM26 (600 kBq). The animals were euthanized by CO_2_ asphyxiation 3 h and 24 h pi immediately before being placed in the camera. Whole body SPECT/CT scans were performed using the Triumph™ Trimodality System (TriFoil Imaging, Inc., Northridge, CA, USA) at the following parameters: CT: 80 mm field of view (FOV), 1.48 magnification, 1 projection, and 512 frames; SPECT: 80 mm FOV and 75A10 collimators (5 pinholes). CT raw files were reconstructed by Filter Back Projection (FBP). SPECT raw data were reconstructed using an ordered Subset Expectation Maximization (OSEM) iterative reconstruction algorithm. SPECT and CT data were analyzed using PMOD v3.508 (PMOD Technologies Ltd., Zurich, Switzerland) and were presented as maximum intensity projections (MIPs).

### 2.7. PET/CT Imaging Using ^55^Co-NOTA-PEG_2_-RM26

Male NOD-SCID mice bearing PC-3 xenografts were intravenously injected with 0.18 nmol (approx. 3 MBq) of ^55^Co-NOTA-PEG_2_-RM26 in the tail vein. At 3 h pi the animals (*n* = 3) were anesthetized using isoflurane and PET/CT scanned using the Siemens Inveon Multimodality preclinical scanner (Siemens Healthcare, Knoxville, USA). At 24 h pi the animals were euthanized by intraperitoneal injection of pentobarbital and PET/CT scanned again. CT parameters were 2 bed positions, 360 projections in 360 degrees' rotation, and bin 4. The PET acquisition times of the static scans were 900 s and 1800 s at 3 h and 24 pi, respectively. The PET data were attenuation corrected using the coregistered CT scan and the sinograms were reconstructed using the OSEM3D-MAP reconstruction algorithm (4 OSEM3D iterations, 16 MAP subsets, and 18 MAP iterations, target resolution 0.8 mm). PET and CT data were analyzed using Inveon Research Workplace (Siemens Healthcare) and presented as MIPs adjusted to display a color scale from 0 to the maximum tumor uptake value.

## 3. Results and Discussion

Prostate cancer is a complex disease with a heterogeneous growth pattern where some patients may not require active treatment, while others need drastic intervention. In view of this biologic and clinical heterogeneity, it is imperative to distinguish between rapidly and slowly progressing disease and to accurately determine the stage of PCa in order to prescribe appropriate treatments. Molecular imaging of GRPR expression can contribute substantially in the initial diagnosis and staging of PCa by noninvasively assessing the lymph node involvement and other metastases and distinguishing low- from high-risk disease. However, imaging of low abdominal lymph node involvement in PCa is challenging and requires the highest possible sensitivity.

In a recent study we have evaluated the effect of macrocyclic chelators on the biodistribution and targeting properties of ^111^In-labeled bombesin antagonist RM26 (^111^In-X-PEG_2_-RM26 (X = NOTA, NODAGA, DOTA, and DOTAGA)) [13]. In addition to the main conclusions related to aspects that underlie the influence of radionuclide-chelator complexes, the study also highlighted the crucial role of radionuclide half-life in high sensitivity molecular imaging and pointed that next day imaging may be preferable due to higher contrast. In this regard, all conjugates evaluated in that study demonstrated significantly higher tumor-to-organ ratios at 24 h pi compared to 4 h pi. Therefore, we have investigated in this project a new probe, ^55^Co-NOTA-PEG_2_-RM26, for next day PET imaging of GRPR expression in PCa that would combine the advantages of later imaging with the high sensitivity and quantitative accuracy of PET.

**Figure 1:**
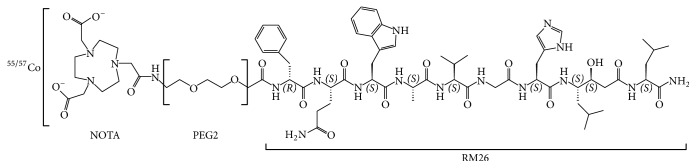
Schematic overview of the structure of ^55/57^Co-NOTA-PEG_2_-RM26.

**Figure 2:**
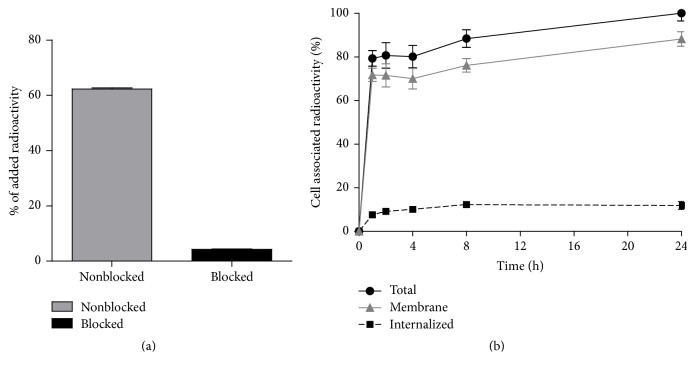
(a) In vitro specificity test of ^57^Co-NOTA-PEG_2_-RM26 binding to GRPR on PC-3 (human prostate cancer) cells. Presaturation of receptors with unlabeled NOTA-PEG_2_-RM26 caused significant (*p* < 0.05) reduction of cell-bound ^57^Co-NOTA-PEG_2_-RM26 radioactivity. (b) Binding and internalization of ^57^Co-NOTA-PEG_2_-RM26 at 37°C by PC-3 cells. Data are normalized to a maximum cell-bound radioactivity and presented as average value from 3 cell dishes ± SD. Not all error bars are visible due to the small standard deviations.

Labeling of NOTA-PEG_2_-RM26 with radiocobalt was performed in acidic conditions that should prevent oxidation of Co(II) by atmospheric oxygen to Co(III) that is common in alkaline solutions [21]. Labeling was successful, with yields exceeding 99% for ^57^Co and 98% for ^55^Co. ^57^Co-NOTA-PEG_2_-RM26 (Figure 1) was characterized by excellent stability (Table 1) and retained the capacity to bind specifically to GRPR-expressing PC-3 cells (Figure 2(a)). Binding of ^57^Co-NOTA-PEG_2_-RM26 to GRPR-expressing PC-3 cells was significantly (*p* < 1.1 × 10^−8^) reduced when presaturating the receptors with a large molar excess of nonlabeled peptide, indicating that binding of ^57^Co-NOTA-PEG_2_-RM26 is receptor-dependent. The binding pattern of ^57^Co-NOTA-PEG_2_-RM26 was characterized by a rapid increase in total cell-associated radioactivity within the first hour of incubation, followed by a slower increase until the 24 h time point (Figure 2(b)). An interesting find was the very low internalized fraction that reached only 12% of cell-associated radioactivity after 24 h incubation (Figure 2(b)). Although a low internalized fraction is expected from antagonists, the internalized fraction for ^57^Co-labeled NOTA-PEG_2_-RM26 was threefold lower compared to the same radioconjugate labeled with ^111^In [7, 13]. This might reflect lower residualizing properties of cobalt-NOTA containing radiocatabolites.

**Figure 3:**
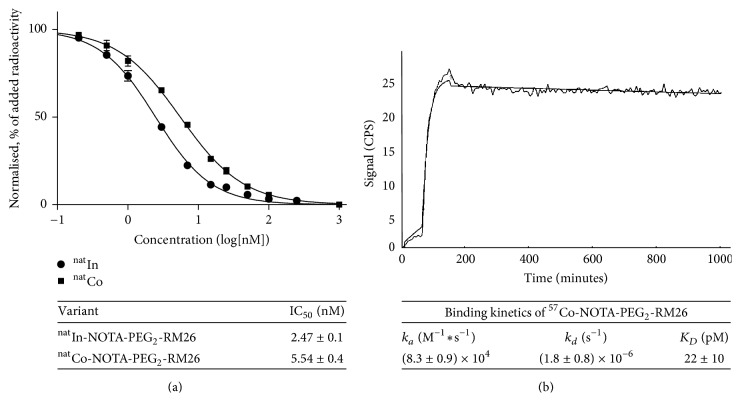
(a) Inhibition of ^125^I-Tyr^4^-BBN binding to PC-3 cells with ^nat^Co-NOTA-PEG_2_-RM26 and ^nat^In⁡-NOTA-PEG_2_-RM26. Data are presented as mean values of three dishes ± SD. (b) LigandTracer sensorgram and fitted binding curve of ^57^Co-NOTA-PEG_2_-RM26 interaction with GRPR-expressing PC-3 cells at room temperature. Uptake curves were recorded at 0.33 and 6 nM. The data were fitted to a one-to-one interaction model.

The binding properties of the peptide loaded with cobalt and, for comparison, indium were evaluated in a competitive binding assay using ^125^I-Tyr^4^-BBN as displacement radioligand (Figure 3). While both IC_50_ values were in the low nanomolar range, the cobalt-loaded analog ^nat^Co-NOTA-PEG_2_-RM26 had two fold higher IC_50_ values (5.5 ± 0.4 nM) compared to the ^nat^In⁡-loaded counterpart (2.5 ± 0.1 nM). These findings are in good agreement with previous results obtained for bombesin and somatostatin analogs, confirming that the net charge of the N-terminus of the conjugate has a strong impact on the binding affinity [12, 13, 22, 23]. In this case, the positive N-terminus charge of indium-NOTA complex resulted in higher affinity, while a neutral charge (cobalt-NOTA complex) decreased the binding affinity. Real-time measurements of ^57^Co-NOTA-PEG_2_-RM26 binding affinity to GRPR-expressing PC-3 cells revealed *K*_*D*_ values in the low picomolar range (22 ± 10 pM) with a rapid binding (association rate of 8.3 × 10^4^  ±  8.7 × 10^3^ 1/M × sec) and slow dissociation (dissociation rate of 1.8 × 10^−6^  ±  8.0 × 10^−7^ 1/sec). One important advantage of using real-time measurements is the ability to detect the time to equilibrium, which eliminates the risk of premature interruption of incubation in other assays. In such a way the intrinsic limitation of the end-point in vitro assays to study high affinity conjugates (*K*_*D*_ determination in saturation assay, IC_50_ determination in inhibition assay) is avoided. In the end-point assays the values of high affinity conjugates (in low picomolar range) are underestimated because of the extremely long time required to reach equilibrium [24]. It should be noted that high affinity binding is a major precondition when aiming for high contrast next day imaging.

**Figure 4:**
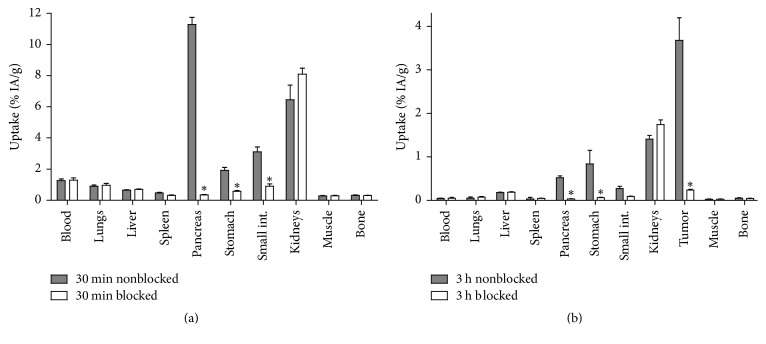
In vivo binding specificity of ^57^Co-NOTA-PEG_2_-RM26 in (a) NMRI mice at 30 min pi and (b) PC-3 xenografted Balb/c nu/nu mice at 3 h pi. The total injected mass of radioconjugate was 45 pmol. All animals in the blocked group were coinjected with 20 nmol of nonlabeled peptide. Data are presented as an average % ID/g and standard deviation for four mice. *∗* stands for significant difference (*p* < 0.05) in uptake of radioactivity in the respective organs between the blocked and nonblocked groups of mice.

The biodistribution of ^57^Co-NOTA-PEG_2_-RM26 in female NMRI mice was characterized by rapid clearance from blood and normal tissues already at 30 min pi (Figure 4(a)). The clearance was predominantly renal with a low kidney retention (6 ± 2% ID/g) and low liver uptake (0.65 ± 0.05% ID/g). Receptor-positive abdominal organs (pancreas, small intestine, and stomach) showed high uptake. In these organs, the accumulated radioactivity was significantly lower in the group of mice coinjected with excess amount of unlabeled peptide indicating specific uptake of ^57^Co-NOTA-PEG_2_-RM26. These results were further confirmed in female Balb/c nu/nu mice bearing subcutaneous PC-3 tumors at 3 h pi where the tumor uptake and the uptake in GRPR-expressing organs were inhibited by coinjection of an excess amount of unlabeled peptide (Figure 4(b)). The accumulation of radioactivity in normal organs and excretory organs was not significantly different upon blocking.

**Figure 5:**
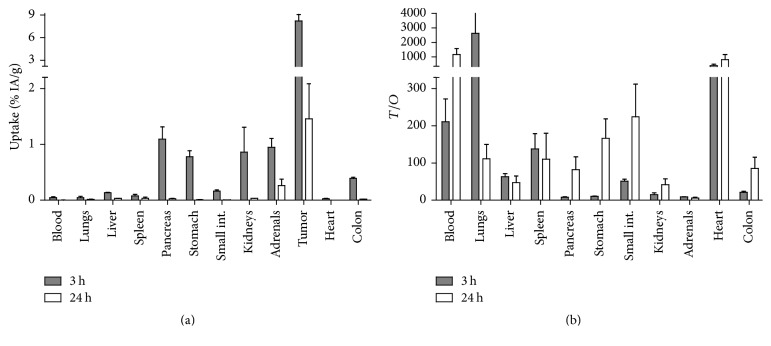
(a) Biodistribution of ^57^Co-NOTA- PEG_2_-RM26 in male NOD-SCID mice bearing PC-3 xenografts at 3 and 24 h pi (total injected mass of 16.8 ± 3.6 pmol). (b) Tumor-to-organ ratios of ^57^Co-NOTA- PEG_2_-RM26 in NOD-SCID mice with subcutaneous PC-3 xenografts. Data are presented as an average value and standard deviation for three mice.

Data concerning the biodistribution of ^57^Co-NOTA-PEG_2_-RM26 over time in PC-3 xenografted mice are presented in Figure 5. The overall biodistribution pattern of the cobalt-labeled peptide and the tumor uptake were in good agreement with the data published for this peptide labeled with indium-111, gallium-68, and fluorine-18 via aluminum fluoride [7, 8, 12, 13]. A surprising finding of this study was the significantly faster whole body and blood clearance of ^57^Co-NOTA-PEG_2_-RM26 compared to the previously evaluated ^68^Ga and ^111^In-labeled NOTA-PEG_2_-RM26 [7]. In this regard, at the 3 h time point the blood levels of radioactivity for ^57^Co-labeled NOTA-PEG_2_-RM26 were 2-fold lower than ^111^In- and 6-fold lower compared to ^68^Ga-labeled analogs. Different clearance rates from circulation for peptides and proteins labeled with different radiometals using the same chelator are a known phenomenon [25–27]. This could be due to different coordination geometries that could affect the binding to blood proteins, therefore modulating the excretion rate. The excretion of radioactivity was predominantly through the kidneys, as the radioactivity uptake in both the liver and gastrointestinal tract was low at all time points.

The rapid clearance of radioactivity from blood (0.05 ± 0.02% ID/g) and normal organs resulted in high tumor-to-organ ratios already at 3 h pi (Figure 5(b)). Remarkably, the tumor uptake exceeded the uptake in all other organs and was 7-fold higher than pancreas, the organ with the second highest uptake at 3 h pi. This discrepancy in retention of radioactivity between xenografts and receptor-positive organs was previously reported for ^68^Ga and ^111^In-labeled NOTA-PEG_2_-RM26 [7] as well as other bombesin analogs [28–30] and could be attributed to interspecies differences between mouse and human GRPR or to receptor density in tissues. The rapid radioactivity washout from normal and receptor-positive organs together with a higher retention of the radioconjugate in tumors resulted in a further increase of tumor-to-organ ratios over time. Therefore, exceptionally high tumor-to-blood, tumor-to-pancreas, tumor-to-stomach, and tumor-to-kidney ratios were achieved at 24 h pi. For comparison, for radiocobalt-labeled bombesin agonist AMBA the tumor-to-blood ratio at this time point was 7-fold lower, tumor-to-kidney 9-fold lower, tumor-to-liver 1.5-fold lower, tumor-to-colon 80-fold lower, and tumor-to pancreas 300-fold lower [14].

**Figure 6:**
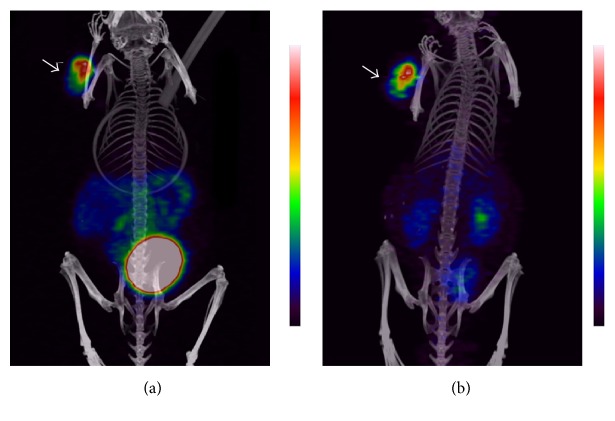
Coronal MIP preclinical PET/CT images showing tracer distribution in PC-3 xenografted NOD-SCID mice. The animals were injected with 0.18 nmol of ^55^Co-NOTA-PEG_2_-RM26 (approx. 3 MBq) and scanned at (a) 3 h and (b) 24 h pi. The tumor is shown by the arrow.

The high potential of radiocobalt-labeled NOTA-PEG_2_-RM26 for imaging of GRPR-expressing tumors was confirmed by *μ*PET/CT images using ^55^Co-NOTA-PEG_2_-RM26 (Figure 6). Similar to the biodistribution results, the predominant radioactivity uptake was seen in tumors. The kidney and urinary bladder uptake could also be visualized at both 3 h and 24 h pi due to the predominant renal excretion of the radiotracer. The SPECT/CT and *μ*PET/CT MIP images together with the biodistribution results suggest 24 h pi as the optimal time point for image acquisition.

**Figure 7:**
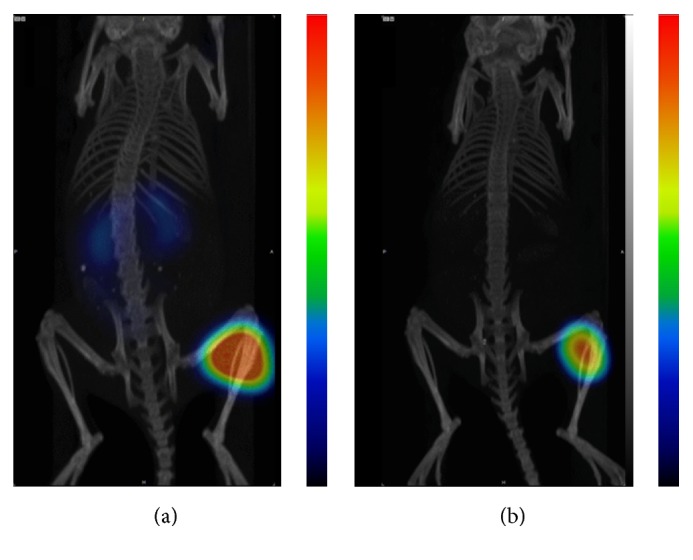
Coronal MIP preclinical SPECT/CT images showing tracer distribution in PC-3 xenografted BALB/C nu/nu mice. The animals used for SPECT camera imaging were injected with 45 pmol of ^57^Co-NOTA- PEG_2_-RM26 (300 kBq) and euthanized at (a) 3 h and (b) 24 h pi.

Cobalt-55 was used in ionic form in several clinical studies as tracer that may reflect calcium (Ca) influx [31–33]. This PET radioisotope has favorable properties for noninvasive imaging: relatively long half-life (17.5 h), production at low-energy cyclotrons available in most PET facilities, and the highest positron abundancy among positron-emitting radiometals with comparable half-life (Table 2). It can be produced by low-energy medical cyclotrons available in most PET facilities and due to the long half-life it can be distributed to distant sites. The production costs of a batch of cobalt-55 should be in the same range as a batch of copper-64. However, until recently radiocobalt was not used for labeling of peptides and proteins for molecular imaging due to undeveloped labeling chemistry. Within the last years several groups reported labeling with radiocobalt isotopes of scaffold proteins affibody molecules DOTA-Z_HER2_ [27] and DOTA-Z_HER1_ [34], GRPR-agonistic BN analog NOTA-AMBA [14], and somatostatin analogs DOTATATE, DOTATOC, and DOTANOC [18, 19]. Both tetra- and tri-aza-chelators demonstrated stable coordination of divalent cobalt. For preclinical development cobalt-55 is rather expensive. Cobalt-57 has half-life of 271.6 days, emits photons with convenient energy (122 keV (86%), 136 keV (10%)), and can be used for preclinical experiments in the same way as iodine-125 is used for development of radiotracers instead of iodine-124 (PET isotope), iodine-123 (SPECT isotope), and iodine-131 (therapeutic isotope). To prove this, we performed *μ*SPECT/CT imaging of GRPR-expressing tumors using ^57^Co-NOTA-PEG_2_-RM26 at 3 h and 24 h pi (Figure 7). Similar to the *μ*PET/CT images and the biodistribution results, the predominant radioactivity uptake was seen in tumors. Traces of radioactivity could also be observed in the kidneys at 3 h pi. At the 24 h pi, due to the fast clearance of radioactivity from normal organs, only the tumor could be visualized.

## 4. Conclusions

We have developed a peptide-based targeting agent labeled with ^55^Co for next day high contrast PET imaging of GRPR-expressing tumors. We have also demonstrated that long lived ^57^Co can be used as a surrogate for ^55^Co in preclinical studies.

## Figures and Tables

**Table 1:** Labeling and stability of ^55/57^Co-NOTA-PEG_2_-RM26.

^55/57^Co-NOTA-PEG_2_-RM26	
Labeling yield for ^57^Co	99.8 ± 0.2%
Labeling yield for ^55^Co	98.6%
Release in the presence of excess EDTA^*∗*^	0.7 ± 0.2%
Release in PBS^*∗*^	0.07 ± 0.11%

^*∗*^Stability of ^57^Co-NOTA-PEG_2_-RM26 was checked after 1 h incubation at room temperature in the presence of 1000x excess of EDTA. Data are presented as average ± standard deviation.

**Table 2:** Long-lived positron-emitting radiometals. Data are taken from [10].

Nuclide (half-life)	Mode of decay	*E*_max_ (keV)	Principal photon emissions, keV (abundance in %)
^55^Co (17.5 h)	*β* ^+^ 76% EC 24%	1498	477 (20.2%), 931 (75%), 1317 (7.1%), 1408 (16.9%)

^64^Cu (12.7 h)	*β* ^+^ 18 *β*^−^ 37% EC 24%	653	1346 (0.5%)

^86^Y (14.7 h)	*β* ^+^ 33% EC 67%	3141	443 (16.9%), 628 (32.6%), 646 (9.2%), 703 (15.4%), 778 (22.4%), 1077 (82.5%), 1153 (30.5%), 1854 (17.2%), 1920 (20.8%)
